# SOCIAL CONNECTEDNESS RESOURCING OF OLDER PEOPLE IN ASSISTED LIVING IN THE UNITED STATES OF AMERICA: A SCOPING REVIEW

**DOI:** 10.1093/geroni/igad104.2381

**Published:** 2023-12-21

**Authors:** Rekha Pudur, Elias Mpofu, Gayle Prybutok

**Affiliations:** University of North Texas, Denton, Texas, United States; University of North Texas, Denton, Texas, United States; University of North Texas, Denton, Texas, United States

## Abstract

Abstract This PRISMA scoping review identifies the emerging evidence on social connectedness resources preferences and priorities of older adults in assisted living facilities. The searches in this scoping review were conducted in EBSCOhost. Eight studies were included from 134 articles published in English from January 2000 – September 2022. There are 2482 older adults from 233 assisted living facilities in the USA. Results organized by themes framed in the World Health Organization’s International Classification of Functioning, Disability, and Health (WHO-ICF) 1 provided evidence that older adults with or aging with or into chronic health conditions preferred facility based on community-based resources. Those from the larger enrollment assisted living facilities and limited physical mobility preferred facility-based resources more than community resources. Residents’ preferences and priorities for social connectedness in facility and community-based activities appear to serve different needs, suggesting that resident facilities should deliberately provide for social needs resources by the context of participation. Further research is needed to profile what residents preferred/prioritized in socially inclusive activities, either facility provided or community-based.

